# Successful implantation of a novel polymer-free everolimus-eluting stent using nitrogen-doped titanium dioxide film with good patency on follow-up angiography: A case report

**DOI:** 10.1097/MD.0000000000029666

**Published:** 2022-07-22

**Authors:** Seok Oh, Myung Ho Jeong, Dae Sung Park, Munki Kim, Jung Ha Kim, Dae Young Hyun, Kyung Hoon Cho, Min Chul Kim, Doo Sun Sim, Young Joon Hong, Ju Han Kim, Youngkeun Ahn

**Affiliations:** a Department of Cardiology, Chonnam National University Hospital, Gwangju, Korea; b Department of Cardiology, Chonnam National University Medical School, Gwangju, Korea; c Cardiovascular Research Center, Chonnam National University Hospital, Gwangju, Korea.

**Keywords:** biomedical technology, coronary artery disease, drug-eluting stents, stents

## Abstract

Despite technological advances in interventional cardiology during the last decades, many concerns remain regarding the narrowing and occlusion of the in-stent area. Particularly, polymer materials pose several problems, including chronic arterial inflammation, impaired arterial healing, and stent thrombosis. To avoid these complications, we invented the TIGEREVOLUTION stent with a cobalt–chromium alloy-based stent platform deposited with N-TiO_2_ film, which has demonstrated good biocompatibility. As this stent is not coated with polymer, it is expected to have decreased risk of stent thrombosis.

**Patient concerns:**

A 62-year-old Korean man visited our department because of angina. We commenced coronary angiography (CAG).

**Diagnosis:**

CAG revealed critical stenosis in the mid-portion of the right coronary artery, with a minimum lumen area of 1.08mm^2^ on optical coherence tomography (OCT).

**Intervention:**

Percutaneous coronary intervention was performed with implantation of a novel 3.5 × 26-mm polymer-free everolimus-eluting stent using nitrogen-doped titanium dioxide film (TIGEREVOLUTION^®^ stent). Post-percutaneous coronary intervention OCT showed good stent expansion and apposition, and the patient was discharged successfully and uneventfully.

**Outcomes:**

Eight months later, follow-up coronary angiography demonstrated good stent patency with no definitive evidence of in-stent restenosis, with thin stent strut coverage demonstrated on OCT.

**Lessons:**

We report the first case of TIGEREVOLUTION stent implantation with follow-up OCT at 8 months.

## 1. Introduction

Atherosclerotic coronary artery disease is a leading cause of mortality, and its gradually increasing prevalence poses a high socioeconomic burden.^[1]^ Patients with unstable or clinically significant atherosclerotic lesions are conventionally treated with percutaneous coronary intervention (PCI), which involves balloon angioplasty and stent implantation.

Since the first-in-human coronary angioplasty performed by Gruntzig in 1977,^[2]^ PCI has substantially evolved with the development of coronary stents and with numerous studies aiming to refine the design, structure, and material of stents. The advent of bare-metal stents (BMSs) substantially reduced the complications associated with coronary angioplasty and, consequently, decreased the rates of both restenosis and coronary artery bypass graft surgery. However, increasing utilization of BMSs led to an increased incidence of in-stent restenosis (ISR), a gradual decrease in the luminal diameter inside the stent strut that occurs several months after PCI, resulting in recurrent angina that requires target lesion revascularization.^[3,4]^ Although the commercial introduction of the first-generation drug-eluting stents (DESs) reduced the many drawbacks associated with BMSs, it also introduced unexpected and new hazards, such as late and very late stent thrombosis (ST), compared with prior treatments. Despite the introduction of high intensity and prolonged treatment with antithrombotics to overcome these problems, prolonged use of antithrombotics causes bleeding complications. A transformative breakthrough in PCI was achieved with the development of second-generation DESs made of biodegradable or more biocompatible polymers with improved drug release formulation, stent platforms, and designs, resulting in decreased ISR, ST, and bleeding complications.^[5,6]^ Nevertheless, as polymer materials have been reported to cause chronic arterial inflammation, endothelial injury, and ST,^[7]^ problems related to polymer-based DESs remain as major concerns.

To overcome these problems, we previously invented a novel drug-coating technique that uses a titanium dioxide (TiO_2_) film and does not require polymer materials.^[8,9]^ By using this technique, we also invented a novel polymer-free everolimus-eluting stent with nitrogen-doped TiO_2_ (N-TiO_2_) film, named the TIGEREVOLUTION^®^ stent.^[8,9]^

We herein report a case of successful implantation of the TIGEREVOLUTION stent.

## 2. Case report

**Figure 1.**
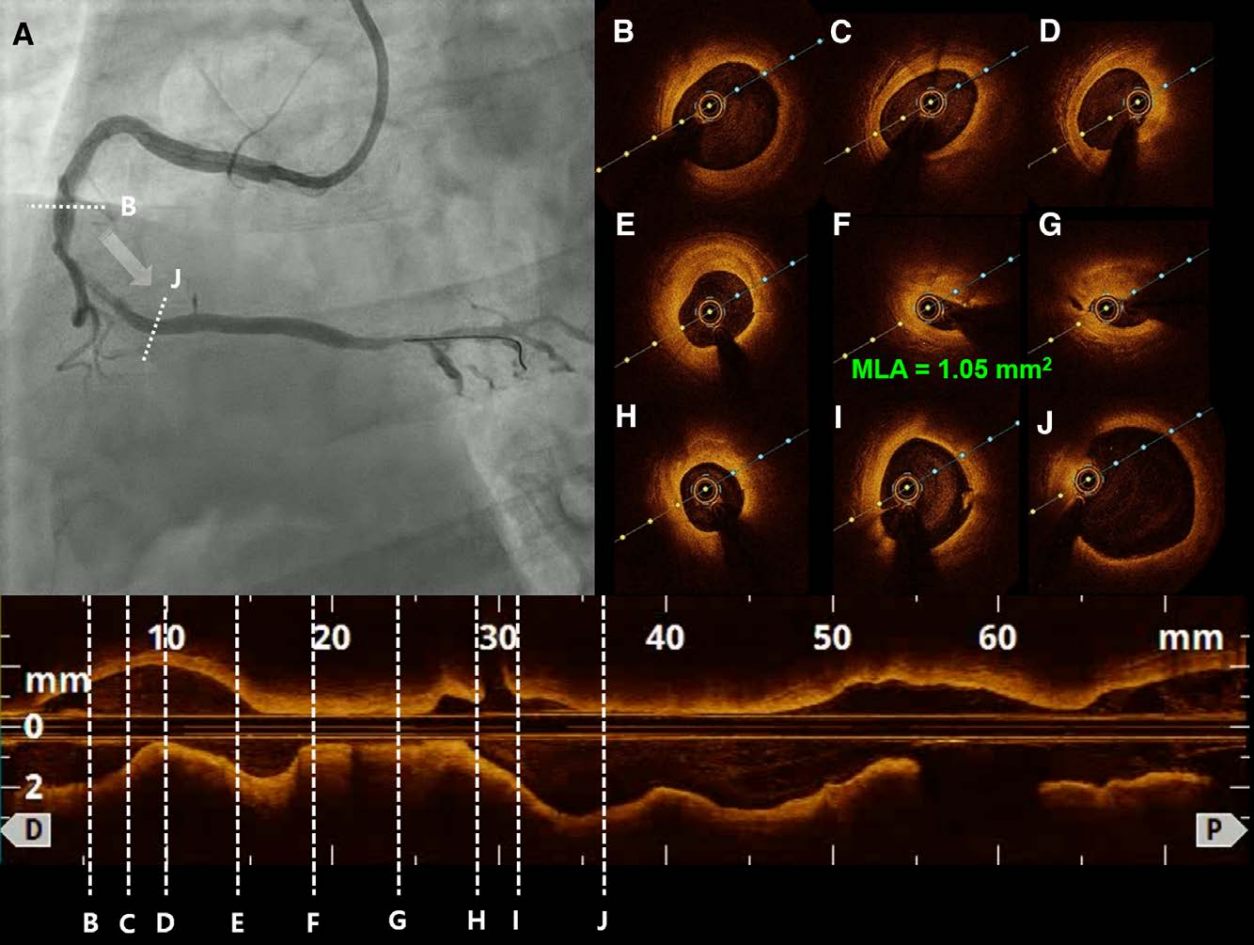
Initial CAG and OCT findings. (A) CAG demonstrates critical stenosis in the middle portion of the right coronary artery. (B–J) OCT using a 2.7-Fr C7 Dragonfly imaging catheter (LightLab Imaging Inc., Westford, MA), performed before percutaneous coronary intervention, demonstrates an MLA of 1.05 mm^2^ with a plaque burden of 86%. CAG = coronary angiography, MLA = minimum lumen area, OCT = optical coherence tomography.

A 62-year-old male patient of Korean ethnicity presented to our tertiary cardiovascular center because of acute chest pain. Coronary angiography (CAG) revealed critical stenosis in the middle portion of the right coronary artery (**Fig. 1A**; Video 1, Supplemental Digital Content, http://links.lww.com/MD/G943). We performed optical coherence tomography (OCT) using a 2.7-Fr C7 Dragonfly imaging catheter (LightLab Imaging Inc., Westford, MA; **Fig. 1B–J**; Video 2, Supplemental Digital Content, http://links.lww.com/MD/G944), which demonstrated a minimum lumen area of 1.05 mm^2^ with a plaque burden of 86% (**Fig. 1F**).

**Figure 2.**
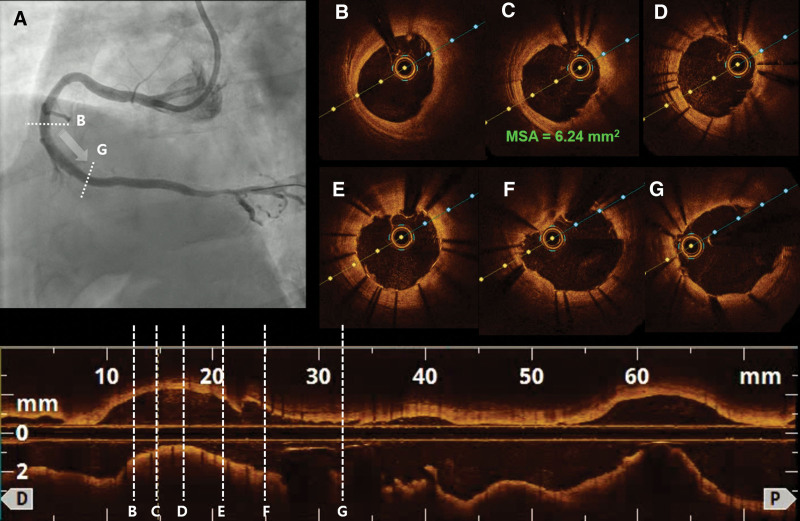
CAG and OCT findings after PCI. (A) After PCI with a novel polymer-free everolimus-eluting stent using N-TiO2 film (TIGEREVOLUTION [TIGER EVerOlimus eLUting stent using TIO2 thiN film], 3.5 × 26 mm; CG Bio, Seongnam, Republic of Korea), CAG shows good results without residual stenosis. (B–G) Post-PCI OCT revealing good stent expansion and apposition, with an MSA of 6.24 mm^2^. CAG = coronary angiography, MSA = minimum stent area, N-TiO2 = nitrogen-doped titanium dioxide, OCT = optical coherence tomography, PCI = percutaneous coronary intervention.

We implanted a novel polymer-free everolimus-eluting stent using N-TiO_2_ film (TIGEREVOLUTION [TIGER EVerOlimus eLUting stent using TiO_2_ thiN film], 3.5 × 26 mm; CG Bio, Seongnam, Republic of Korea). After PCI with the TIGEREVOLUTION stent, repeat CAG showed good antegrade flow without residual stenosis (**Fig. 2A**; Video 3, Supplemental Digital Content, http://links.lww.com/MD/G945). Thereafter, post-PCI OCT was performed, which demonstrated a well-expanded and apposed stent strut, with a minimum stent area of 6.24 mm^2^ (**Fig. 2C**; Video 4, Supplemental Digital Content, http://links.lww.com/MD/G946). The patient showed improvement in clinical symptoms and signs and was discharged from the hospital successfully and uneventfully.

**Figure 3.**
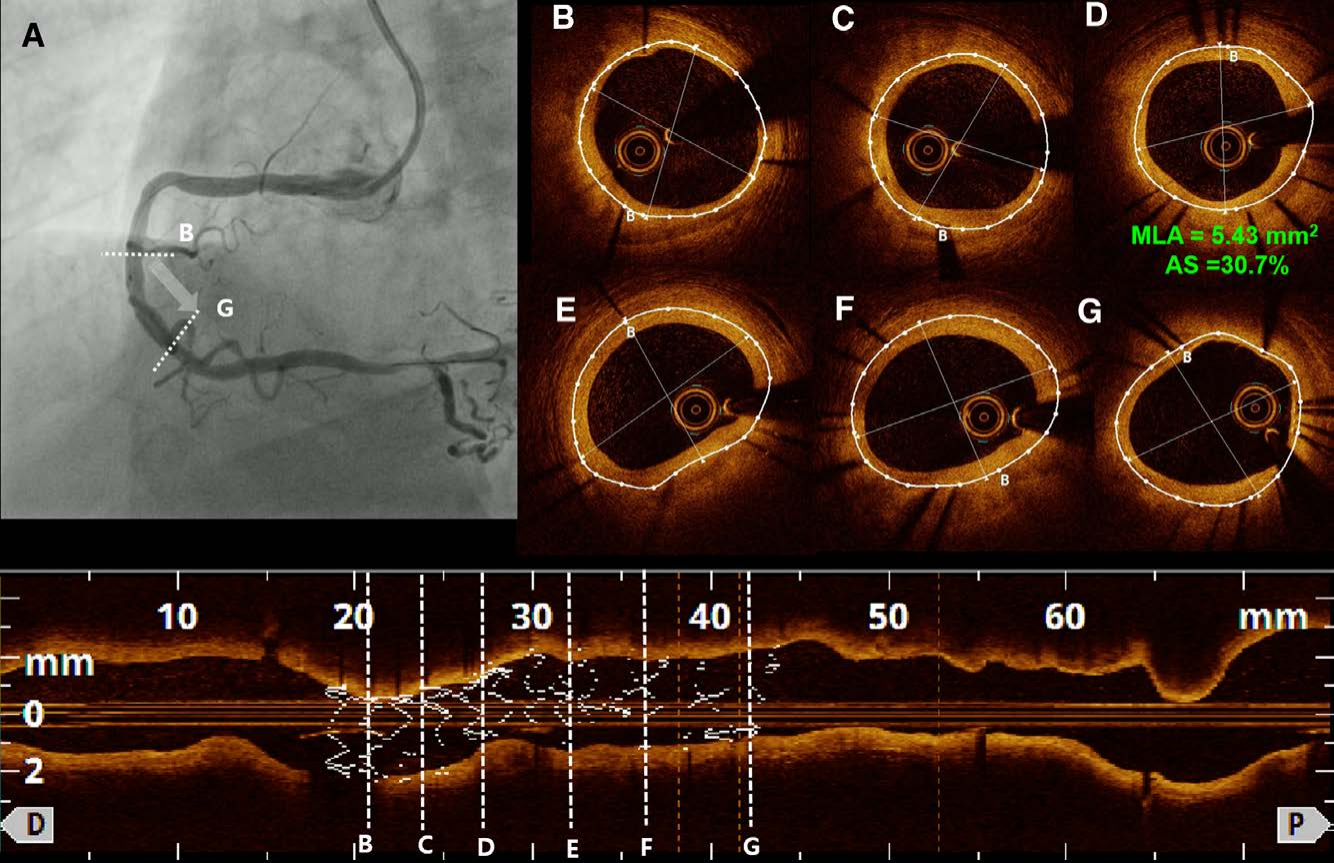
CAG and OCT findings 8 months after PCI. (A) At 8 months after PCI, follow-up CAG showing good stent patency with no definitive evidence of in-stent restenosis. (B–G) OCT shows thin stent strut coverage with an MLA of 5.43 mm2 and an AS of 30.7%. AS = area stenosis, CAG = coronary angiography, MLA = minimum lumen area, OCT = optical coherence tomography, PCI = percutaneous coronary intervention.

Eight months later, the patient underwent follow-up CAG for the evaluation of stent patency. The follow-up CAG demonstrated good stent patency with no definitive evidence of ISR (**Fig. 3A**; Video 5, Supplemental Digital Content, http://links.lww.com/MD/G947). On OCT, thin stent strut coverage (neointimal hyperplasia) was noted (**Fig. 3B–G**; Video 6, Supplemental Digital Content, http://links.lww.com/MD/G948). The minimum lumen area was 5.43 mm^2^, and the percentage area stenosis in this segment was estimated to be 30.7% (**Fig. 3D**). In cross-sectional OCT images analyzed at 1-mm longitudinal intervals, the mean area stenosis was estimated to be 28.29%.

Our study was conducted according to the ethical standards of the Declaration of Helsinki. The patient’s clinical information and data are available from the electronic medical records of Chonnam National University Hospital. This study was exempted from review by the institutional review board of our hospital.

## 3. Discussion

The last half century has seen remarkable advances in interventional cardiology. Since their introduction in the 1980s, coronary stents have undergone revolutionary changes. When BMS was introduced as an alternative to balloon angioplasty, the incidence of ISR increased, which led to target lesion revascularization rates of up to 20% to 30%.^[10,11]^ Thereafter, the first-generation DES, which is composed of a stainless-steel platform, antiproliferative agents, and polymer coating, was developed as a replacement for BMS. Several studies demonstrated that DES considerably reduced the ISR-related revascularization rates.^[12,13]^ However, new concerns emerged about late ST due to delayed in-stent reendothelialization.^[14,15]^ In other words, as endothelial cells were exposed to blood hinder platelet-to-platelet adhesion and aggregation, late ST can occur with incomplete or delayed reendothelialization.^[16,17]^ To overcome this major problem, the second-generation DES was invented with a thinner stent strut and enhanced polymer biocompatibility for drug release.

Despite transformative technological advances in interventional cardiology during the last decades, many concerns remain about narrowing and occlusion of the in-stent area. In particular, polymer materials pose several problems, including chronic arterial inflammation, impaired arterial healing, and ST.^[7]^ For these key reasons, the next-generation stent materials were developed with emphasis on the absence of polymers. For example, bioresorbable vascular scaffolds (BVSs) that are degradable to nontoxic compounds were developed to overcome the limitations of permanent metallic DESs. Nonetheless, BVSs raised concerns, including higher incidences of periprocedural myocardial infarction and scaffold thrombosis than those reported with metal-based DESs, in several randomized clinical trials.^[18,19]^ The short- to mid-term outcomes in the Absorb BVS program, including increased rates of scaffold thrombosis and other concerning features, led to the withdrawal of the Absorb BVS, a poly-lactate-based BVS introduced into the US market in 2018.^[20]^ Although BRSs based on other materials have been developed, the complications remain.

The TIGEREVOLUTION stent was developed to solve the problems associated with polymer materials. Its stent platform was designed with a cobalt–chromium alloy with very thin stent strut (70 µm) and coating (50 nm). It was subjected to a delicate manufacturing process with laser cutting (Rofin; Starcut, Hamburg, Germany) and ultrasonic cleaning using ethanol, acetone, and distilled water.^[8]^ Furthermore, N-TiO_2_ film was deposited onto this platform using plasma-enhanced chemical vapor deposition.^[8,9]^ This stent is a promising alternative to polymer-based DESs and has demonstrated good adhesion, biocompatibility, and mechanical stability.^[21,22]^ The drug release rate of the TIGEREVOLUTION stent has been shown to be comparable to that of a commercial durable-polymer everolimus-eluting stent (XIENCE Prime^®^; Abbott Vascular, Santa Clara, CA).^[23,24]^

We previously investigated the efficacy of a polymer-free everolimus-eluting stent with N-TiO_2_ film deposition in a pig coronary model of ISR with a 4-week follow-up.^[24]^ Furthermore, we also performed an OCT-based preclinical comparative study between the TIGEREVOLUTION and XIENCE Alpine^®^ stents, which demonstrated comparable efficacy and safety outcomes.^[8]^ On the basis of this preclinical evidence, the use of the TIGEREVOLUTION stent in human beings was approved by the Korean Food and Drug Administration on July 31, 2020. This case report is the first to describe the implantation of a TIGEREVOLUTION stent in the human coronary artery.

## 4. Conclusions

We herein report the first case of successful implantation of a novel polymer-free everolimus-eluting stent using N-TiO_2_ film, named the TIGEREVOLUTION stent.

## Author contributions

**Conceptualization:** Seok Oh, Dae Young Hyun, Kyung Hoon Cho, Doo Sun Sim

**Methodology**: Seok Oh, Kyung Hoon Cho

**Investigation**: Seok Oh

**Resources**: Seok Oh, Dae Young Hyun, Kyung Hoon Cho, Min Chul Kim, Doo Sun Sim, Young Joon Hong, Ju Han Kim, Youngkeun Ahn, Myung Ho Jeong

**Data curation:** Seok Oh, Kyung Hoon Cho

**Writing—original draft preparation:** Seok Oh

**Writing—review and editing:** Seok Oh, Dae Sung Park, Munki Kim, JungHa Kim, Dae Young Hyun, Kyung Hoon Cho, Min Chul Kim, Doo Sun Sim, Young Joon Hong, Ju Han Kim, Youngkeun Ahn, Myung Ho Jeong

**Project administration**: Seok Oh, Kyung Hoon Cho

## Supplementary Material


